# Effects of Sound-Pressure Change on the 40 Hz Auditory Steady-State Response and Change-Related Cerebral Response

**DOI:** 10.3390/brainsci9080203

**Published:** 2019-08-16

**Authors:** Eishi Motomura, Koji Inui, Yasuhiro Kawano, Makoto Nishihara, Motohiro Okada

**Affiliations:** 1 Department of Neuropsychiatry, Mie University Graduate School of Medicine, Tsu 514-8507, Japan; 2 Department of Functioning and Disability, Institute for Developmental Research, Aichi Human Service Center, Kasugai 480-0392, Japan; 3 Multidisciplinary Pain Center, Aichi Medical University, Nagakute 480-1195, Japan

**Keywords:** ASSR, change detection, magnetic field, N1m, sound pressure

## Abstract

The auditory steady-state response (ASSR) elicited by a periodic sound stimulus is a neural oscillation recorded by magnetoencephalography (MEG), which is phase-locked to the repeated sound stimuli. This ASSR phase alternates after an abrupt change in the feature of a periodic sound stimulus and returns to its steady-state value. An abrupt change also elicits a MEG component peaking at approximately 100–180 ms (called “Change-N1m”). We investigated whether both the ASSR phase deviation and Change-N1m were affected by the magnitude of change in sound pressure. The ASSR and Change-N1m to 40 Hz click-trains (1000 ms duration, 70 dB), with and without an abrupt change (± 5, ± 10, or ± 15 dB) were recorded in ten healthy subjects. We used the source strength waveforms obtained by a two-dipole model for measurement of the ASSR phase deviation and Change-N1m values (peak amplitude and latency). As the magnitude of change increased, Change-N1m increased in amplitude and decreased in latency. Similarly, ASSR phase deviation depended on the magnitude of sound-pressure change. Thus, we suspect that both Change-N1m and the ASSR phase deviation reflect the sensitivity of the brain’s neural change-detection system.

## 1. Introduction

The brain’s ability to automatically respond to sensory changes in the environment accelerates the execution of appropriate behaviors. The early stage of neural processing can be recorded by electroencephalography (EEG) and by magnetoencephalography (MEG) with high temporal resolution. An abrupt change in a continuous sound evokes a clear cerebral response, peaking at approx. 100–180 ms following a change onset in sound frequency [1,2,3,4], sound intensity [1,5,6,7,8,9,10], sound location [1,11,12,13], or timbre [14].

This cerebral response was based on comparisons between preceding and novel stimuli with some form of sensory memory [12,15]. The magnitude of this cerebral response depended on the degree of the sound-feature change [1,7,13]. Thus, the cerebral response seems to be a type of auditory change-related cerebral response (called “Change-N1”, and its magnetic counterpart “Change-N1m”). Change-N1 is also evoked by an abrupt decrease (dec-Change-N1) [8,9,16], as well as an abrupt increase (inc-Change-N1) in sound pressure.

The auditory steady-state response (ASSR) is a neural oscillation that is phase-locked to a repeated sound stimulus. It can be recorded by EEG and MEG. The ASSR becomes stable at approx. 200 ms after onset. In humans, the ASSR can be recorded with maximum amplitudes when stimuli are presented at 40 Hz. The ASSR has been implicated in the functional integrity of the local neural network for auditory processing. Several studies reported that the 40 Hz ASSR phase varies after stimulus changes, such as a noise [17], interaural-phase difference [18,19], frequency [20], and a gap [21].

Ross suggested that ASSR phase deviation might be a type of auditory-change response [19]. However, it remains unclear whether ASSR phase deviation could be affected by the degree of the sound-feature change in the same way as the change-related cerebral response described in previous studies [1,7,13]. In the present study, using click-train sounds, we simultaneously recorded the 40 Hz ASSR and Change-N1m evoked by abrupt change in sound pressure, and we investigated the effect of the magnitude of the sound-pressure change on these two cerebral responses.

## 2. Materials and Methods

### 2.1. Subjects

The experiment was performed with 10 healthy volunteers (2 females and 8 males; mean age: 35.6 years; 22–54 years) with normal hearing. All subjects had no history of substance abuse, neurological, otolaryngologic, or psychiatric disease. They were medication-free. The study was approved in advance by the Ethics Committee of the National Institute for Physiological Sciences, Okazaki, Japan (18A036). Written consent was obtained from all of the subjects.

### 2.2. Sound Stimuli

The subjects were instructed to watch a silent movie, ignoring the sound stimuli delivered through ear pieces (E-A-Rtone 3A, Aero, Indianapolis, IN, USA). The presented stimulus was a train of 1 ms clicks at 40 Hz. The control stimulus was 1000 ms in length and 70 dB in sound-pressure level. The change stimuli (deviant) were also 1000 ms long; the first 500 ms was identical to the control stimulus, which was followed without a blank by similar 500 ms click-trains whose sound pressure was different. Therefore, the sound pressure of the deviants changed abruptly at the midpoint. The sound-pressure changes of the deviants were −5, −10, −15, 5, 10, or 15 dB; thereby, there were 6 deviants. The trail–trial interval was 1500 ms. All sound stimuli were presented randomly at an even probability in a session. The time necessary for recording was 22–25 min.

### 2.3. MEG Recording

Magnetic responses were recorded with a helmet-shaped 306-channel MEG system (Vector-view; ELEKTA Neuromag, Helsinki, Finland) comprised of 102 triple-sensor elements in a magnetically shielded room. Each sensor element consisted of two orthogonal planar gradiometers and one magnetometer coupled to a multi-superconducting quantum interference device (SQUID), providing 3 independent measurements of the magnetic fields per sensor. In this study, we analyzed MEG signals recorded using 204 planar-type gradiometers. Signals were recorded with a bandpass of 0.1–330 Hz and digitized at 2000 Hz. Trials with noise larger than 3000 fT/cm were excluded from averaging. For each stimulus, at least 120 trials are averaged.

### 2.4. Dipole Source Modeling

**Figure 1:**
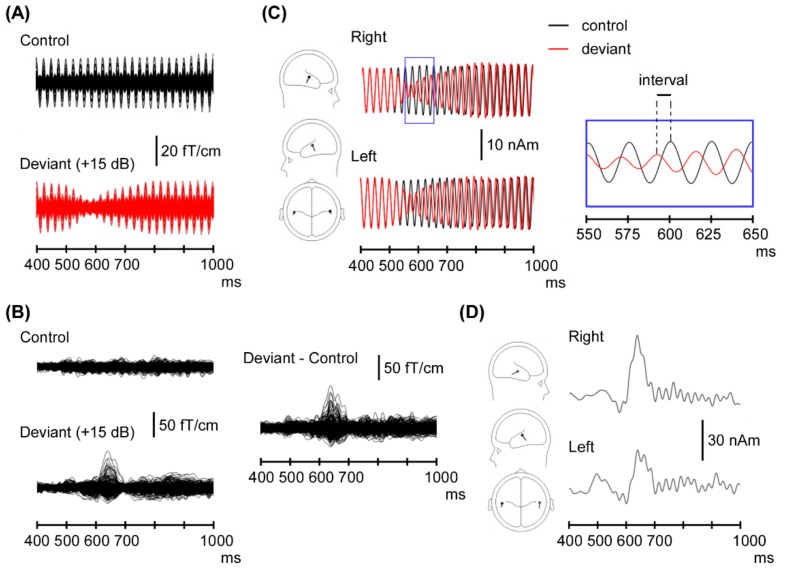
Magnetic responses and source-strength waveforms. Data from a representative case. Superimposed magnetoencephalography (MEG) waveforms recorded from 204 gradiometers with a bandpass filter of (**A**) 35–45 Hz for the auditory steady-state response (ASSR) and (**B**) 1–35 Hz for the magnetic response peaking at approximately 100–180 ms (called “Change-N1m”). (**C**,**D**) Estimated dipole models and source-strength waveforms. Part of waveforms with expanded time axis is shown.

We analyzed the recorded MEG waveforms using brain electric-source analysis (BESA, version 6.0, GMbH, Munich, Germany). First, we estimated 2-equivalent current dipole (ECD) models (1 in each temporal region) for cerebral responses. A spherical head model was used for the dipole source analysis. Figure 1 shows the dipole source-modeling procedure.

For the 40 Hz ASSR, bandpass filters were 35–45 Hz. The baseline was a 100 ms period before sound onset. The first 500 ms was identical among all stimuli. In order to create an ECD model, dipoles were estimated across the time window from 400 to 500 ms in the control condition because the ASSR was stable during this period. The obtained dipole model was applied to all ASSR waveforms in each subject, and source-strength waveforms were used for analysis. For the Change-N1m, bandpass filters were 1–35 Hz. The baseline was a 100 ms period before the sound change. We obtained Change-N1m using subtracting waveforms for the control stimulus from that for the +/− 15 dB deviant-stimulus conditions. The measurement interval of the Change-N1m peak was approx. 100–180 ms after the change onset in a continuous sound. A 20 ms period around the Change-N1m peak was used for dipole analysis. Similar to ASSR analysis, the estimated dipole models for +/− 15 dB deviant-stimulus conditions were applied to the remaining of the increase (+10 and +5 dB) and decrease (−10 and −5 dB) deviant-stimulus conditions in each subject.

### 2.5. Data Analysis

In the ASSR, the time interval between peaks to each click, which was stimulus-locked with each click, showed transient deviation in the deviant condition (Figure 1C). Although it is well known that ASSR amplitude changes after the change onset, the ASSR phase deviation was used as an index in the present study. We defined phase deviation as the time difference between peak latencies for each click between the control- and deviant-stimulus conditions. When analyzing Change-N1m values, to minimize the problem due to a baseline shift, we determined amplitude as the amplitude between the peak of Change-N1m and the polarity-reversed peak at an earlier latency, as in our previous studies [1,8,10]. If a positive peak was not detected, the voltage at 40 ms after the change onset was measured. The head coordination system was set by the nasion and two reference points anterior to the ear canals. The *x*-axis was fixed at the preauricular points and defined the right and left directions. The *y*-axis was defined as the anterior–posterior directions through the nasion. The *z*-axis was defined as the superior–posterior directions.

The statistical significance of the source location was assessed by discriminant analysis using the *x, y*, and *z* coordinates as variables. For analyses of the ASSR phase shift and Change-N1m values, we conducted multivariate analysis of variance (MANOVA) with repeated measures of within-subject factors (increase/decrease in sound pressure, left/right hemisphere, and degrees of change), and the Bonferroni–Dunn test as a post hoc comparison. *P*-values < 0.05 were considered significant.

## 3. Results

**Figure 2:**
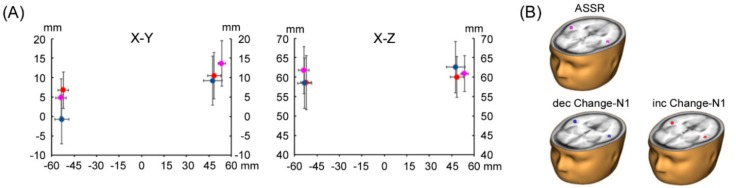
Dipole location. Mean locations of estimated current dipole (ECD) (**A**) demonstrated and (**B**) superimposed on a standard MR image. Error bars indicate 95% confidence intervals for each dipole.

Reliable ECDs for dec-Change-N1m were not estimated in two subjects because of a low signal/noise (S/N) ratio. In one subject, the 40 Hz ASSR phase was not stable in the 100 ms duration before the change onset in the control stimulus. These three MEG responses were excluded from further analysis. The ECDs of the ASSR, inc-Change-N1ms, and dec-Change-N1ms were estimated to be located at the auditory cortex on both hemispheres. The location of ECDs responsible for Change-N1m and ASSR are shown in Figure 2. The results of discriminant analysis revealed that the ECD location did not significantly differ between the ASSR and Change-N1m for all conditions (*p* = 0.35–0.90).

### 3.1. ASSR Phase Deviation

**Figure 3:**
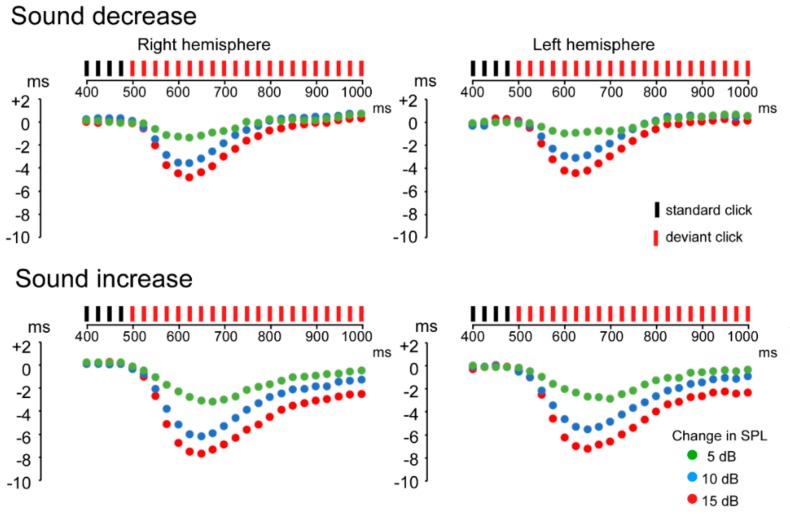
Phase-difference time courses of each response to the deviant click from control click. Group means (dots) of peak-latency interval from control condition for each click are plotted. Compared to the control stimulus, all deviant-stimulus conditions transiently showed a short interval. Deviation depended on the magnitude of sound-pressure change.

In the deviant-stimulus conditions, peak-latency interval for each click became shorter after the change in sound pressure, and subsequently returned to the steady state. As shown in Figure 3, phase deviation in deviant-stimulus conditions reached a maximum at around 100–200 ms after the sound pressure’s change onset. As the magnitude of change increased, deviation was prolonged.

**Figure 4:**
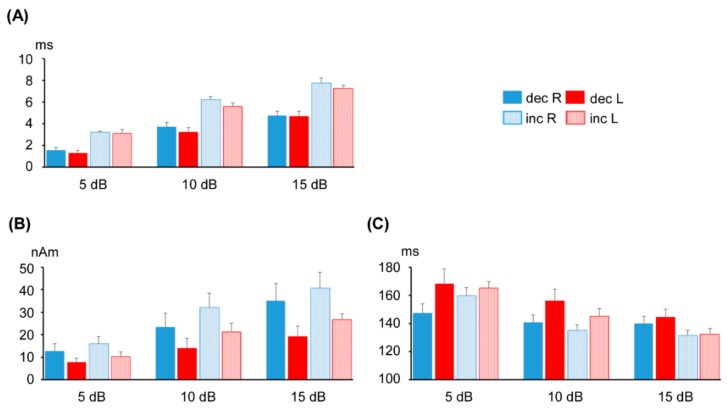
Effects of magnitude of sound-pressure change. (**A**) Mean maximum time interval of peak latency in the ASSR, and (**B**) mean peak amplitude and (**C**) latency of Change-N1m. nAm: Nanoampere-meters; error bars: Standard mean errors.

As shown in Figure 4A, the results of the repeated MANOVA revealed a significant difference in the maximum interval of the phase deviation between increase/decrease (F(1,8) = 32.8, *p* = 4.4 × 10^−5^) and the degree of change in sound pressure (F(2,16) = 118.8, *p* = 2.5 × 10^−10^), but not between hemispheres (F(1,8) = 1.8, *p* = 0.22). The Bonferroni–Dunn test showed that the maximum interval was significantly greater in the ±15 dB deviant condition than in the ±10 dB (*p* = 9.7 × 10^−5^) and ±5 dB (*p* = 7.0 × 10^−6^) deviant conditions. The maximum interval was greater in the ±10 dB deviant condition than in the ±5 dB (*p* = 2.3 × 10^−5^) deviant condition.

### 3.2. Change-N1m Values

As shown in Figure 4B, the results of the repeated MANOVA showed that the Change-N1m peak amplitude significantly differed between increase/decrease (F(1,8) = 8.6, *p* = 0.019) and degrees of change (F(2,16) = 23.6, *p* = 1.6 × 10^−5^). In addition, amplitude was significantly greater for the right hemisphere (F(1,8) = 9.8, *p* = 0.014). The Bonferroni–Dunn test showed that the Change-N1m peak amplitude was significantly greater in the ±15 dB deviant condition than in the ±10 dB (*p* = 0.009) and ±5 dB (*p* = 0.001) deviant conditions, and significantly greater in the ±10 dB deviant condition compared to the ±5 dB (*p* = 0.022) deviant condition.

As shown in Figure 4C, the MANOVA results showed that the peak latency of Change-N1m was significantly shorter for the right hemisphere (F(1,8) = 10.2, *p* = 0.013). Peak latency also significantly differed among degrees of change (F(2,16) = 29.8, *p* = 4.1 × 10^−6^), but not between increase and decrease (F(1,8) = 2.7, *p* = 0.14). The Bonferroni–Dunn test showed that peak latency was significantly shorter in ±15 dB deviant condition than in the ±10 dB (*p* = 0.02) and ±5 dB (*p* = 4.7 × 10^−4^) deviant conditions. The difference between the latter two conditions was also significant (*p* = 0.006). There was an interaction between increase/decrease and degrees of change (F(2,16) = 3.9, *p* = 0.04). Peak latency was shorter in the increase compared to the decrease deviant condition in the ±15 dB (*p* = 0.02) and ±10 dB (*p* = 0.04) conditions.

## 4. Discussion

Our present findings clarified the effect of an abrupt change in sound pressure on the ASSR phase and the Change-N1m. The ASSR phase alternated after the change onset and subsequently returned to the steady state in all of the deviant conditions. The time course of the ASSR phase elicited by an abrupt change in this study is congruent with previous studies [17,19,21]. Regardless of increase/decrease in sound pressure, both the ASSR phase deviation and the Change-N1m values (peak amplitude and latency) depended on deviance in sound pressure from the preceding sound.

As in earlier MEG studies, ASSR dipoles were estimated to be in the auditory cortex [17,18,19,20,21]. The dipoles for Change-N1m were estimated in similar areas (Figure 2). Change-N1m is elicited by any type of auditory changes, including sound onset [7]. Previous MEG studies reported that all estimated dipoles were located in the lateral part of the transverse gyrus, and their location did not differ [2,11,13]. A functional magnetic-resonance-imaging study revealed lacking tonotopic organization in lateral regions of the auditory cortex [22]. Combined with our MEG studies, we speculate that Change-N1m reflects processes for detecting changes of any type in the surrounding environment rather than the processing of basic sound features. In the present study, dipole locations were not different between ASSR and Change-N1ms. This finding was incongruent with previous studies showing that ASSR sources were located more medially than N1m. However, comparison of the ECD location between ASSR and Change-N1m was not the main purpose of the present study.

An abrupt change in sound feature induces a cerebral response that is based on a comparison between preceding and current stimuli. This auditory-change-detection system was investigated by using Change-N1 responses. As the degree of the sound-pressure change from the baseline increased, Change-N1 amplitude increased and peak latency decreased [1,7]. The present results confirmed this. Similarly, we observed that, as the degree of sound-pressure decrease increased, dec-Change-N1m amplitude increased and its peak latency decreased. Interestingly, ASSR phase deviation showed similar behaviors against the magnitude of the sound-pressure change. One hypothesis for ASSR generation is that it is produced by the superimposition of a midlatency response (MLR) for each click sound [23]. The amplitude and latency of the MLR components are affected by sound intensity [24], but the transient ASSR phase deviation in the present result could not be explained by this superimposition hypothesis. Both ASSR phase deviation and Change-N1m values depended on the degree of change in sound pressure. Thus, we consider that both are transient cerebral responses to the event in the surrounding environment. Considering that the maximum phase deviation of the ASSR and the peak of Change-N1m were detected in the same time range (about 100–200 ms after the change onset), both responses could be produced by a similar neural circuit for auditory-change detection.

Although the stimulation paradigms differ, these findings are congruent with those in mismatch negativity (MMN) studies [25,26]. Thus, regardless of increase or decrease in sound pressure, both Change-N1 and MMN seemed to depend on deviance from preceding sound pressure. Traditional MMN is elicited by comparison between deviant stimuli and a repeated standard stimulus in an oddball paradigm. However, multifeature MMN paradigms have been reported [27,28,29], which has merit in that MMN to different deviant stimuli could be recorded in a single session and in a relatively short time, similar to the present stimulus paradigm. However, such an MMN paradigm with sound-to-sound intervals seems to not be suitable to record ASSR.

It is well known that each hemisphere is optimized for various tasks for processing sensory information, and both hemispheres have complementary roles. Although ASSR phase deviation did not show a significant difference between hemispheres in this study, Change-N1m amplitude was larger in the right hemisphere than in the left, which is congruent with a previous study [2]. In addition, Change-N1m peak latency was shorter in the right hemisphere. The present results confirmed that the right hemisphere plays an important role in detecting auditory environmental changes.

The 40 Hz ASSR is useful for assessing the ability to integrate sensory information with high test–retest reliability [30]. Kwon et al. first reported that subjects with schizophrenia showed diminished ASSR power and a delayed phase of the ASSR oscillation [31]. One of the possible underlying mechanisms is that gamma-amino butyric acid (GABA) inhibitory interneurons modulate the generation and synchronization of neural oscillation. Deficits in the ASSR linked to abnormal GABA transmission in schizophrenia have been reported [32,33]. We suggest that a 40 Hz click-train stimulus with an abrupt change in sound pressure could be useful to simultaneously assess the sensitivity of auditory-change detection in addition to the functional integrity of the local neural network. Change-N1m could also be measured by this method. The stimulus paradigm used in the present study makes it possible to multidimensionally assess cognitive deficits in psychiatric disorders.

## 5. Conclusions

We investigated the relationship between two automatic cerebral responses, ASSR and Change-N1m, and effects of the magnitude of sound-pressure change in a train of 40 Hz click sounds. The results show that both the ASSR phase deviation and Change-N1m reflect the automatic cerebral process of change detection. However, there are some limitations in the present study. Although we used dipoles in the auditory cortex for both ASSR and Change-N1m, it is known that subcortical or frontal regions contribute to ASSR and N1 as well, which might affect the results. Furthermore, in order to validate the present results, we need to adopt other methods, such as time-frequency analysis that addresses intertrial power and neural-phase-locking underlying evoked responses [34], as well as ASSR [17].
